# Gender differences in factors associated with smartphone addiction: a cross-sectional study among medical college students

**DOI:** 10.1186/s12888-017-1503-z

**Published:** 2017-10-10

**Authors:** Baifeng Chen, Fei Liu, Shushu Ding, Xia Ying, Lele Wang, Yufeng Wen

**Affiliations:** School of Public Health, Wannan Medical College, 22 West wenchang Road, Wuhu, Anhui Province 241002 China

**Keywords:** Smartphone addiction, Problematic smartphone use, Sleep quality, Depression, Anxiety

## Abstract

**Background:**

Smartphones are becoming increasingly indispensable in everyday life for most undergraduates in China, and this has been associated with problematic use or addiction. The aim of the current study was to investigate the prevalence of smartphone addiction and the associated factors in male and female undergraduates.

**Methods:**

This cross-sectional study was conducted in 2016 and included 1441 undergraduate students at Wannan Medical College, China. The Smartphone Addiction Scale short version (SAS-SV) was used to assess smartphone addiction among the students, using accepted cut-offs. Participants’ demographic, smartphone usage, and psycho-behavioral data were collected. Multivariate logistic regression models were used to seek associations between smartphone addiction and independent variables among the males and females, separately.

**Results:**

The prevalence of smartphone addiction among participants was 29.8% (30.3% in males and 29.3% in females). Factors associated with smartphone addiction in male students were use of game apps, anxiety, and poor sleep quality. Significant factors for female undergraduates were use of multimedia applications, use of social networking services, depression, anxiety, and poor sleep quality.

**Conclusions:**

Smartphone addiction was common among the medical college students investigated. This study identified associations between smartphone usage, psycho-behavioral factors, and smartphone addiction, and the associations differed between males and females. These results suggest the need for interventions to reduce smartphone addiction among undergraduate students.

## Background

Smartphones are no longer considered simply as “mobile phones”, but real-time information providers and powerful portable computers. This is because these portable devices provides a variety of functions such as phone, camera, multimedia player, internet browser, navigation system, and e-mail service as well as facilitate social networking and game playing. As smartphones were developed in recent years, China became the largest market for these devices, and the market continues to grow at an astonishing pace. According to China’s 39th Statistical Report on Internet Development, 695 million Chinese individuals were accessing the internet through smartphones as of December 2016 [1]. Despite the advantages associated with smartphone use, such as enhanced social networking, increased productivity, and more dynamic and immediate ways of living and working, prior research suggests that many people overuse their phones in ways that interfere with their daily lives [2, 3], safety [4], health [5–7], and physical health-related problems such as blurred vision and pain in the wrists or neck [8]. Moreover, smartphone overuse may lead to mental or behavioral problems, such as changes in attitude towards school or work, reduction of social interaction in real life, and disorders [9].

Griffiths operationally defined technological addiction as a type of non-chemical behavioral addiction that involves human-machine interaction [10]. Gambling addiction, which is the most well-known behavioral addiction, has been categorized within “substance-related and addictive disorders” in the latest version of Diagnostic and Statistical Manual of Mental Disorders (DSM-5) [11]. Internet gaming disorder has also been listed in the research criteria of the DSM-5 [11]. Several self-reported questionnaires have been developed to assess problematic smartphone use or “addiction” in recent years [12–14]. Generally, smartphone addiction consists of four main components: compulsive phone use, behaviors such as repeated checking for messages or updates; tolerance, longer and more intense bouts of use; withdrawal, feelings of agitation or distress without the phone; and functional impairment, interference with other life activities and face to face social relationships [15]. All these are very similar to the characteristics of internet addiction [16].

To date, little is known about factors associated with problematic or addictive smartphone usage. In a community study of young adults, high mobile phone usage was associated with subsequent stress, sleep difficulties, and depression [16]. In another study, 362 employed adults were tested for associations between age, self-esteem, extraversion, emotional stability, depression on the one hand, and impulse control in relation to different levels of addictive and problematic mobile phone use on the other [17], and the study found that age, depression, and extraversion were predictors of higher scores on level of problematic mobile phone use. In another study conducted on smartphone users, anxiety and depression were found to be higher in groups that over-used smartphones than in normal user groups [7]. Similarly, it has been reported that problematic use of the internet is associated with sleep problems, such as subjective insomnia and poor sleep quality [18, 19].

When compared to older social groups, undergraduate students are more likely to make heavy use of smartphones and hence are potentially vulnerable to smartphone addiction [20]. As young people, undergraduates are digital natives who have grown up in the smartphone age and have integrated this appliance into their lifestyle [21]. Tao et al. [22] assessed problematic mobile phone use in a large random sample of Chinese adolescents in 2012 and reported that the prevalence of problematic mobile phone use among Chinese adolescents was 26.2%. Another study reported that the prevalence of problematic smartphone use among Chinese undergraduates was 21.3% [23]. Although a previous study indicated differences in smartphone addiction by gender [24], there are limited data in the literature regarding gender differences in the prevalence of smartphone addiction or the associated factors in a large population of Chinese undergraduate students. The present study was the first to investigate the relationship between smartphone usage and psycho-behavioral–related variables associated with smartphone addiction in male and female undergraduate students.

## Methods

### Participants and procedures

This cross-sectional study was conducted in September 2016 to analyze factors associated with smartphone addiction among medical college students at Wannan Medical College, China. The study was reviewed and approved by the Ethics Committee of Wannan Medical College. Written informed consent was obtained from all participants prior to completion of the questionnaire survey. Participation in the study was not compulsory: the students were informed that they were not obliged to participate, that all responses were anonymous, and that they were free to refuse to answer any questions. A total of 1556 students were randomly selected as the study sample, and 115 (7.4%) were excluded from the analysis because they failed to complete the questionnaires in its entirety. Respondents completed a validated screening tool for smartphone addiction and provided information on socio-demographics, smartphone use characteristics, depression, anxiety, and sleep quality. All questionnaires were in the self-report format. To avoid typing errors, we entered information into all questionnaires using a double-entry strategy in EpiData, version 3.1 (EpiData Association, Odense, Funen, Denmark).

### Measurement

The information collected included basic socio-demographic data, such as gender, age, academic year, residence (urban/rural), and monthly cost of living. Specific information regarding smartphone use included the age at which they obtained their first smartphone, the most-often-used functions (phone call/message, games, video watching/listening to music, social networking services, internet surfing/e-book reading, and others), daily smartphone use time, frequency of replacement of their smartphones, and monthly smartphone bill.

Smartphone addiction was measured using the Smartphone Addiction Scale short version (SAS-SV). The SAS-SV is a validated scale that contains 10 items rated on a dimensional scale (1 “strongly disagree” to 6 “strongly agree”) [14]. The total score ranges from 10 to 60, with the highest score representing the maximum presence of “smartphone addiction” in the past year. The original SAS-SV showed content and concurrent validity and internal consistency (Cronbach’s alpha: 0.91). Smartphone addiction cut-off values of ≥ 31 and ≥ 33 for male and female participants, respectively, were applied as suggested by Kwon et al. [14].

Anxiety symptoms were measured with the 20-item Self-Rating Anxiety Scale (SAS) developed by Zung [25]. A Chinese version of the SAS was used in the present survey. The scale has been proven to correspond well with the assessment of anxiety and has been found to be reliable in epidemiological surveys of Chinese populations [26, 27]. Each item contains four options that describe how often the respondents ha each feeling in the previous week on a four-point Likert scale ranging from “none or a little of the time (less than once day)” (coded as 1) to “most or all the time (5–7 days)” (coded as 4). The total score on the SAS was calculated, with higher scores indicating a higher degree of anxiety. According to previous studies of the Chinese population [26, 27], anxiety was defined in this study by a total raw score above 40.

Depressive symptoms were measured with the 20-item Chinese version of the Center for Epidemiologic Studies Depression Scale (CES-D) [28]. This scale is widely used for assessment of depressive symptoms in normal and clinical samples from different ages, genders, and nationalities [29, 30]. Participants were asked to rate the occurrence of each symptom in the previous week on a scale ranging from 1 (rarely) to 4 (most of the time). Total scores ranged from 20 to 80, with higher scores indicating higher levels of depressive symptoms. Scores of 36 or higher are considered indicative of depression. The Chinese version of the CES-D exhibits high degrees of reliability and validity [31].

Sleep quality was assessed using Pittsburgh Sleep Quality Index (PSQI) [32], which is a widely used 19-item self-report questionnaire that measures subjective sleep quality. The 19 items are grouped into scores with seven components: subjective sleep quality, sleep latency, sleep duration, sleep efficiency, sleep disturbances, use of sleep medication, and daytime dysfunction. The scores of these components are added to a global PSQI score with a range of 0 to 21, with higher scores indicating worse sleep quality. PSQI scores above 5 were taken as abnormal. The Chinese version of PSQI developed by Liu et al. [33] is widely used in different populations, and it has good reliability for assessment of undergraduate students [34, 35].

### Statistical analysis

Descriptive analysis was used to examine the demographic and psychological behavior characteristics of the study participants. Univariate analysis by chi-square tests and multivariate analysis by binary logistic regression analysis were used to identify factors related to smartphone addiction in different genders. As suggested by Hosmer, Lemeshow and Sturdivant (2013, pages 89–94), variables that had a significance level of *p* < 0.20 in the univariate analysis were considered potential factors for inclusion in the multivariate models [36]. Final multivariate models were determined by performing stepwise backward elimination to remove covariates not significant at the *p* < 0.05 level. The adjusted odds ratios (ORs) and 95% confidence intervals (CIs) were reported. Data entry was done in Epi Info 3.1. The database was then exported to SPSS 20.0 (SPSS Inc., Chicago, IL, USA) for analysis. Values of *p* < 0.05 were considered statistically significant.

## Results

### Gender-related differences in basic characteristics of the study participants

The final sample included 1441 undergraduate students. Out of the 1441 smartphone users, 696 (48.3%) were male, while 745 (51.7%) were female. They were aged 17– 26 years (mean age, 19.72 ± 1.43 years). The results indicated that smartphone addiction was present in 429 (29.8%) of the 1441 participants, and the prevalence of smartphone addiction was 30.3% in males, and 29.3% in females. Table 1 presents the gender differences in the basic characteristics of the study participants.

**Table 1:** Basic gender differences in characteristics of the study participants [n (%)]

	Total (*N* = 1441)	Male (*n* = 696)	Female (*n* = 745)	*p*
Age (years)				
≤ 19	664 (46.1)	306 (44.0)	358 (48.1)	.120
≥ 20	777 (53.9)	390 (56.0)	387 (51.9)	
Residential source				
Rural	928 (64.4)	469 (67.4)	459 (61.6)	.022
Urban	513 (35.6)	227 (32.6)	286 (38.4)	
Monthly cost of living				
≤ 800 RMB	494 (34.3)	212 (30.5)	282 (37.9)	.008
800–1200 RBM	714 (49.5)	371 (53.3)	343 (46.0)	
≥ 1200 RMB	233 (16.2)	113 (16.2)	120 (16.1)	
Most personally relevant smartphone function				
Phone calls/Text message	505 (35.0)	235 (33.9)	270 (36.2)	.000
Smartphone gaming	60 (4.2)	49 (7.0)	11 (1.5)	
Multimedia applications (Watching videos/Listening to music)	230 (16.0)	108 (15.4)	122 (16.3)	
Social networking services	483 (33.5)	208 (29.9)	275 (36.9)	
Internet surfing/reading	92 (6.4)	57 (8.1)	35 (4.7)	
Others	71 (4.9)	39 (5.6)	32 (4.3)	
Smart phone addiction				
Negative	1012 (70.2)	485 (69.7)	527 (70.7)	.662
Positive	429 (29.8)	211 (30.3)	218 (29.3)	
Sleep quality				
Good sleep quality	929 (64.5)	467 (67.1)	462 (62.0)	.044
Poor sleep quality	512 (35.5)	229 (32.9)	283 (38.0)	
Anxiety				
Negative	1301 (90.3)	607 (87.2)	694 (93.2)	.000
Positive	140 (9.7)	89 (12.8)	51 (6.8)	
Depressive				
Negative	1076 (74.7)	501 (72.0)	575 (77.2)	.023
Positive	365 (25.3)	195 (28.0)	170 (22.8)	

### Univariate analysis of factors associated with smartphone addiction between genders

Table 2 presents the univariate analyses of potential factors related to smartphone addiction. Older age, playing of smartphone games, smartphone media applications, poor sleep quality, depression, and anxiety were associated with smartphone addiction in males. In females, older age, higher monthly cost for living, less use of phone calling/messaging, use of social networking service applications, poor sleep quality, depression, and anxiety were associated with smartphone addiction.

**Table 2:** Univariate analysis of the factors associated with smartphone addiction between the two genders [n(%)]

	Males				Females			
	Smartphone addiction positive	Smartphone addiction negative	OR (95% CI)	*p*	Smartphone addiction positive	Smartphone addiction negative	OR (95% CI)	*p*
Age (years)								
≤ 19	80(26.1)	226(73.9)	Ref.		92(25.7)	266(74.3)	Ref.	
≥ 20	131(33.6)	259(66.4)	1.43 (1.03-1.99)	.034	126(32.6)	261(67.4)	1.40 (1.02-1.92)	.040
Residential source								
Rural	141(30.1)	328(69.9)	Ref.		127(27.7)	332(72.3)	Ref.	
Urban	70(30.8)	157(69.2)	1.04 (0.74-1.46)	.835	91(31.8)	195(68.2)	1.22 (0.88-1.68)	.226
Monthly cost for living								
≤ 800 RMB	60(28.3)	152(71.7)	Ref.		74(26.2)	208(73.8)	Ref.	
800–1200 RBM	109(29.4)	262(70.6)	1.05 (0.73-1.53)	.783	96(28.0)	247(72.0)	1.09 (0.77-1.56)	.625
≥ 1200 RMB	42(37.2)	71(62.8)	1.50 (0.92-2.43)	.101	48(40.0)	72(60.0)	1.87 (1.19-2.94)	.006
Phone calls/Text message								
Not main function	144(31.2)	317(68.8)	Ref.		163(34.3)	312(65.7)	Ref.	
Main function	67(28.5)	168(71.5)	0.88 (0.62-1.24)	.459	55(20.4)	215(79.6)	0.49 (0.35-0.70)	.000
Smartphone gaming								
Not main function	188(29.1)	459(70.9)	Ref.		216(29.4)	518(70.6)	Ref.	
Main function	23(46.9)	26(53.1)	2.16 (1.20-3.88)	.009	2(18.2)	9(81.8)	0.53 (0.11-2.49)	.631
Multimedia applications (Watching videos/Listening to music)								
Not main function	189(32.1)	399(67.9)	Ref.		174(27.9)	449(72.1)	Ref.	
Main function	22(20.4)	86(79.6)	0.54 (0.33-0.89)	.014	44(36.1)	78(63.9)	1.46 (0.97-2.19)	.071
Social networking services								
Not main function	139(28.5)	349(71.5)	Ref.		109(23.2)	361(76.8)	Ref.	
Main function	72(34.6)	136(65.4)	1.33 (0.94-1.88)	.107	109(39.6)	166(60.4)	2.18 (1.58-3.00)	.000
Internet surfing/reading								
Not main function	198(31.0)	441(69.0)	Ref.		211(29.7)	499(70.3)	Ref.	
Main function	13(22.8)	44(77.2)	0.66 (0.35-1.25)	.198	7(20.0)	28(80.0)	0.59 (0.25-1.38)	.217
Others								
Not main function	200(30.4)	457(69.6)	Ref.		211(29.6)	502(70.4)	Ref.	
Main function	11(28.2)	28(71.8)	0.90 (0.44-1.84)	.768	7(21.9)	25(78.1)	0.67 (0.28-1.56)	.348
Sleep quality								
Good sleep quality	98(21.0)	369(79.0)	Ref.		98(21.2)	364(78.8)	Ref.	
Poor sleep quality	113(49.3)	116(50.7)	3.67 (2.61-5.16)	.000	120(42.4)	163(57.6)	2.73 (1.98-3.78)	.000
Anxiety								
Negative	165(27.2)	442(72.8)	Ref.		187(26.9)	507(73.1)	Ref.	
Positive	46(51.7)	43(48.3)	2.87 (1.82-4.51)	.000	31(60.8)	20(39.2)	4.20 (2.34-7.56)	.000
Depressive								
Negative	127(25.3)	374(74.7)	Ref.		142(24.7)	433(75.3)	Ref.	
Positive	84(43.1)	111(56.9)	2.23 (1.57-3.16)	.000	76(44.7)	94(55.3)	2.47 (1.73-3.52)	.000

### Multivariate logistic analysis of factors associated with smartphone addiction

Multivariate binary logistic regression models were used to identify the factors associated with smartphone addiction in males and females, separately. For the 696 male students, the following variables were significant factors associated with smartphone addiction: smartphone games (aOR = 2.27; 95% CI: 1.17 – 4.42); PSQI (aOR = 3.19; 95% CI: 2.23 – 4.58); and SAS (aOR = 1.78; 95% CI: 1.09 – 2.89). For the 745 female students, the following variables were significant factors associated with smartphone addiction: multimedia applications (aOR = 2.22; 95% CI: 1.37 – 3.59); social networking applications (aOR = 2.63; 95% CI: 1.81 – 3.81); PSQI (aOR = 2.12; 95% CI: 1.50 – 2.99); SAS (aOR = 2.31; 95% CI: 1.18 – 4.51); and SDS (aOR = 1.84; 95% CI: 1.21 – 2.79). The corresponding results are presented in Tables 3 and 4.

**Table 3:** Multivariate logistic analysis of factors associated with smartphone addiction in males

	aOR (95% CI)	*p*
Main function is smartphone gaming (vs. main function is not smart phone gaming)	2.27 (1.17-4.42)	.015
Poor sleep quality (vs. good sleep quality)	3.19 (2.23-4.58)	.000
Anxiety positive (vs. anxiety negative)	1.78 (1.09-2.89)	.020

Notes: *aOR* djusted Odds Ratio*, CI* Confidence Interval*.* Multivariate binary logistic regression model adjusted for age, residential, and monthly cost

**Table 4:** Multivariate logistic analysis of factors associated with smartphone addiction in females

	aOR (95% CI)	*p*
Main function is multimedia applications (vs. main function is not multimedia applications)	2.22 (1.37-3.59)	.001
Main function is social networking services (vs. main function is not social networking services)	2.63 (1.81-3.81)	.000
Poor sleep quality (vs. good sleep quality)	2.12 (1.50-2.99)	.000
Anxiety positive (vs. anxiety negative)	2.31 (1.18-4.51)	.015
Depressive positive (vs. depressive negative)	1.84 (1.21-2.79)	.004

Notes: *aOR* Adjusted Odds Ratio*, CI* Confidence Interval*.* Multivariate binary logistic regression model adjusted for age, residential, and monthly cost

## Discussion

The prevalence of smartphone addiction in this study (29.8%), as measured with the same SAS, was higher than that in previous studies conducted in South Korea [14] and in Western Europe [37, 38]. The prevalence of smartphone addiction in junior high school students in South Korea was 24.8% [14], while the prevalence rates of smartphone addiction in university students and staff in Spain and Belgium were 12.8% and 21.5%, respectively [37]. The prevalence of smartphone addiction in a sample from a Swiss vocational school was 16.9% [38]. Measurements carried out with different scales in a study in China showed that the prevalence of smartphone-associated problems in undergraduates was 21.3% [23]. Another study reported a 26.2% prevalence level among Chinese adolescents [22], while a 19.1% prevalence has been linked to high-risk smartphone users among Chinese international students in Korea [39]. These discrepancies could be due to the different instruments and classification methods used, and also differences among the participants in the different studies. Yet, the high prevalence rate identified in the current study is an indication of potential public health concern posed by smartphone use among students at the medical college studied.

The results of this study showed no significant gender differences in the prevalence of smartphone addiction (30.3% in males, 29.3% in females, *p* > 0.05). This is similar to the results obtained in some previous investigations [8]. However, some studies have reported that female participants have a higher prevalence of smartphone addiction than males [7, 24, 40]. A study conducted in South Korea suggested that the female participants were more aware of their addiction based on their higher self-reporting scores [41]. Indeed, Demirci reported that the mean SAS score of female students was significantly higher than that of male students [7]. In the present study, how smartphones were used differed between the two genders. Male students were more likely to play games, watch mobile phone videos, and listen to music, whereas female students were more inclined to use the mobile phone communication functions and social networking services. A study carried out by Roberts et al. [42] found that the most problematic applications are voice calls, text messages, and social networks. For females, the cell phone is a means of social contact, in which messaging and social networks play prominent roles, while for males, a more diversified type of usage was observed, involving text messages, voice conversations, and gaming applications [24]. There is still a need for further studies to unravel the inconsistent prevalence of smartphone addiction in males and females.

There was some evidence that smartphone overuse was associated with various psychological and behavioral problems, such as depression, anxiety, and sleep disturbance. However, the nature of the association was unclear. Smartphone use could function as an experiential avoidance strategy to deflect aversive emotional content [43]. It has been found that depressed individuals use their smartphones as a coping strategy to deal with their negative emotions [6]. However, other evidence suggests that excessive smartphone use can trigger psychopathology and sleep disturbances [17, 44]. Excessive smartphone use at night could keep one up late, thus impairing sleep and influencing stress and depression [44]. It has been reported that smartphone overuse may lead to depression and/or anxiety, which can in turn result in sleep problems [7]. Other evidence suggests a bidirectional relationship, whereby excessive smartphone use drives psychopathology, and psychopathology further drives problematic use [45, 46]. The findings of the present study are consistent with those of previous studies with respect to the over-use of smartphones and its relationship to depression, anxiety, and poor sleep quality.

In the present study, some smartphone applications and psycho-behavioral factors were associated with smartphone addiction. Playing smartphone games was a predictor of smartphone addiction for males, whereas use of multimedia and social networking applications were predictors for females. Although the gender difference may only reflect preferences for smartphone apps, the findings indicate the need for gender-targeted prevention and intervention strategies to reduce smartphone addiction. Regarding psycho-behavioral factors, poor sleep quality and anxiety were associated with smartphone addiction in both males and females, but depression was only related in females. It is probable that depression was more prevalent in female students than male students in the present study, as the gender imbalance in depression is described as one of the most robust findings in epidemiological research [47]. Biological and psychosocial factors are proposed to justify the more common onset of depression in females. The effect of sex steroids on the maturating hypothalamic-pituitary-adrenal axis might increase female sensitivity to stress, whereas androgens appear to play a protective role in males [48]. Psychosocial factors include gender differences in stress coping, gender-specific expectations and differences in social cognitive function, with females presenting a greater sensitivity to rejection [49].

Despite the large sample size, the present study has several limitations that should be taken into consideration when interpreting the results. First, the data were cross-sectional, which limits the ability to draw causal inferences, particularly those concerning direction of association between smartphone addiction and risk factors of psychological behavior. A second limitation is the fact that the participants were all medical college students; this may limit the generalization of the present results to other at-risk groups. Thirdly, functional impairments such as blurred vision and pain in the wrists or necks of participants were not investigated here. Thus, generalizing these results to a clinical setting might prove difficult. Lastly, there may be risks and protective factors beyond those measured in the present study, some of which may drive overlap between the measured variables.

## Conclusions

The present study found widespread smartphone addiction among medical college students, which suggests that smartphone addiction has become a public health issue in China. The study also found that playing games on smartphones predicted addiction for male students, whereas uses of multimedia and social networking applications were predictors for females. This study suggests that smartphone addiction may result in psychological and behavioral problems, such as depression, anxiety, and poor sleep quality, and females who scored at the addiction level were more likely to be depressed than males. Considering the gender differences in smartphone addiction highlighted by the present study, targeted prevention and intervention strategies based on a multi-component strategy to reduce this behavioral problem are recommended.
